# Supplementary material for: Spatio-temporal patterns of the crustacean demersal fishery discard from the south Humboldt Current System, based on scientific observer program (2014–2019)
Source: PLoS One. 2023 Feb 27;18(2):e0281932. doi: 10.1371/journal.pone.0281932 (PMC9970059; doi:10.1371/journal.pone.0281932)
Supplement: S1 File — (DOCX) [file pone.0281932.s001.docx]

Supplementary material 1. List of all taxonomic groups identified as discards of the Chilean crustacean demersal fisheries, operating between 2014 and 2019.

**PONE-D-22-26514R1, DOI: 10.1371/journal.pone.0281932**

| Taxonomic group | Scientific name | Abbreviation | FRO(%) |
| --- | --- | --- | --- |
| Teleostei | *Merluccius gayi* | MerGay | 94.970 |
| Teleostei | *Hippoglossina macrops* | HippoMacrops | 60.205 |
| Malacostraca | *Platymera gaudichaudii* | PlatyGaud | 57.245 |
| Malacostraca | *Cancer porteri* | CanPorteri | 54.485 |
| Elasmobranchii | *Coelorinchus aconcagua* | CoelAcag | 33.964 |
| Malacostraca | *Pleuroncodes monodon* | PleuMono | 19.742 |
| Malacostraca | *Cervimunida johni* | CervJohni | 18.629 |
| Teleostei | *Epigonus crassicaudus* | EpiCras | 17.516 |
| Mollusca | *Muusoctopus eicomar* | MuusoEic | 16.025 |
| Elasmobranchii | *Aculeola nigra* | AculNigra | 13.176 |
| Teleostei | *Coelorinchus chilensis* | CoeloChile | 12.976 |
| Elasmobranchii | *Psammobatis scobina* | PsamScob | 12.575 |
| Elasmobranchii | *Centroscyllium granulatum* | CentroGranu | 10.527 |
| Malacostraca | *Heterocarpus reedi* | HeteReedi | 9.103 |
| Malacostraca | *Libidoclaea granaria* | LibGran | 8.547 |
| Teleostei | *Genypterus maculatus* | GenyMacu | 7.367 |
| Elasmobranchii | *Bythaelurus canescens* | BythaCane | 7.300 |
| Elasmobranchii | *Zearaja chilensis* | ZearChile | 6.499 |
| Agnatha | *Eptatetrus polytrema* | EptatPoly | 5.008 |
| Teleostei | *Prolatilus jugularis* | ProlaJug | 3.984 |
| Teleostei | *Guttigadus kongi* | GuttKongi | 3.939 |
| Malacostraca | *Haliporoides diomedeae* | HaliDiom | 3.272 |
| Malacostraca | *Pterygosquilla armata* | PterygArm | 3.116 |
| Mollusca | *Dosidicus gigas* | DosiGigas | 3.027 |
| Elasmobranchii | *Psammobatis rudis* | PsamRud | 2.916 |
| Mollusca | *Muusoctopus longibrachus* | MuusLong | 2.760 |
| Elasmobranchii | *Centroscyllium nigrum* | CentroNigrum | 2.515 |
| Malacostraca | *Lophorochinia parabranchia* | LophoParab | 2.226 |
| Elasmobranchii | *Bathyraja peruana* | BathPeru | 2.003 |
| Anthozoa | *Hormathia pectinata* | HormPect | 2.003 |
| Teleostei | *Bassanago nielseni* | BassNiel | 1.870 |
| Teleostei | *Nezumia pulchella* | NezuPul | 1.469 |
| Elasmobranchii | *Apristurus nasutus* | AprisNasu | 1.113 |
| Elasmobranchii | *Psammobatis* sp. | PSAMMSP | 1.046 |
| Teleostei | *Sebastes oculatus* | SebaOcul | 0.957 |
| Teleostei | *Genypterus chilensis* | GenyChile | 0.868 |
| Elasmobranchii | *Hexanchus griseus* | HexaGriseus | 0.824 |
| Elasmobranchii | *Discopyge tschudii* | DiscoTsch | 0.824 |
| Elasmobranchii | *Gurgesiella furvescens* | GurgFurv | 0.824 |
| Mollusca | *Opisthoteuthis brunni* | OpistBrun | 0.824 |
| Echinodermata | Asteroidea spp. | AsterSpp. | 0.779 |
| Teleostei | *Paralichthys microps* | ParaMicr | 0.779 |
| Elasmobranchii | *Deania calcea* | DeaCalcea | 0.734 |
| Elasmobranchii | *Torpedo tremens* | TorpTrem | 0.712 |
| Elasmobranchii | *Apristurus brunneus* | AprisBrun | 0.601 |
| Mollusca | Gastropoda | Gastropod | 0.579 |
| Teleostei | *Macruronus magellanicus* | MacrurMage | 0.556 |
| Teleostei | *Nezumia pudens* | NezPud | 0.556 |
| Elasmobranchii | *Bathyraja griseocauda* | BathyGrise | 0.512 |
| Elasmobranchii | *Dipturus trachyderma* | DiptTrach | 0.490 |
| Demospongia | Demospongia unident | SpongiaSp | 0.490 |
| Teleostei | *Helicolenus lengerichi* | HELICOLENG | 0.467 |
| Teleostei | *Genypterus blacodes* | GenyBlac | 0.401 |
| Teleostei | *Trachirincus villegai* | TRACHVILL | 0.378 |
| Elasmobranchii | *Centroselachus crepidater* | CentroCrep | 0.312 |
| Teleostei | *Aphos porosus* | APHPORO | 0.312 |
| Elasmobranchii | *Rajella nigerrima* | RajeNige | 0.312 |
| Teleostei | *Trachyrincus helolepis* | TrachyHel | 0.267 |
| Elasmobranchii | *Bathyraja albomaculata* | BathyAlbo | 0.267 |
| Teleostei | *Salilota australis* | SaliAust | 0.223 |
| Mollusca | *Robsonella fontaniana* | ROBSFFONTA | 0.223 |
| Echinodermata | *Hippasteria hyadesi* | HippHyad | 0.223 |
| Mollusca | *Argobuccinum argus* | ArgAgus | 0.200 |
| Malacostraca | *Lithodes turkayi* | LithTurk | 0.200 |
| Elasmobranchii | *Hydrolagus macrophthalmus* | HydroMacrop | 0.200 |
| Elasmobranchii | *Bathyraja multispinis* | BathyMulti | 0.200 |
| Teleostei | *Coryphaenoides armatus* | CoryArmatus | 0.178 |
| Teleostei | *Macrourus carinatus* | MacroCari | 0.178 |
| Teleostei | *Notacanthus sexspinis* | NotaSex | 0.178 |
| Mollusca | *Bathybembix humboldti* | BathyHumb | 0.178 |
| Teleostei | *Coryphaenoides delsolari* | CorypDels | 0.156 |
| Teleostei | *Trachurus murphyi* | TRACHMURP | 0.156 |
| Teleostei | *Beryx splendens* | BerySple | 0.156 |
| Anthozoa | Escleractinas | Esclera | 0.134 |
| Teleostei | *Alepocephalus* sp. | AlepoSp | 0.134 |
| Anthozoa | Sciphozoa | SCIPHO | 0.134 |
|  | Unidentified taxa | Otras.especies | 0.111 |
| Teleostei | *Bajacalifornia megalops* | BajacaMega | 0.111 |
| Teleostei | *Paralabrax humeralis* | ParalHum | 0.111 |
| Malacostraca | *Pagurus imarpe* | PaguIma | 0.111 |
| Teleostei | *Ophichthus* sp. | OphiSp | 0.089 |
| Echinodermata | *Sterechinus agassizii* | SterAgas | 0.089 |
| Echinodermata | Echinoidea | ECHINO | 0.089 |
| Elasmobranchii | *Etmopterus granulosus* | EtmoGran | 0.067 |
| Teleostei | *Brama australis* | BRAMASTR | 0.067 |
| Elasmobranchii | *Sympterygia lima* | SympLima | 0.067 |
| Malacostraca | *Munidopsis* spp. | MunidSpp | 0.045 |
| Echinodermata | *Myxoderma qawashqari* | MyxQawa | 0.045 |
| Teleostei | *Congiopodus peruvianus* | CONGPRU | 0.045 |
| Echinodermata | *Porania* spp. | PORASP | 0.045 |
| Mollusca | *Toradores filippovae* | TODAFIL | 0.045 |
| Malacostraca | Unidentified Malacostraca | JAIBANI | 0.022 |
| Elasmobranchii | *Centroscymnus cryptacanthus* | CENTROCRYP | 0.022 |
| Malacostraca | *Uroptychus milnedwardsi* | UropMiln | 0.022 |
| Teleostei | *Idiacanthus* sp. | IdiacSp | 0.022 |
| Malacostraca | *Projasus bahamondei* | ProjBah | 0.022 |
| Elasmobranchii | Unidentified *Raja* | RAJASI | 0.022 |
| Teleostei | *Aristostomias lunifer* | AristLun | 0.022 |
| Teleostei | *Thyrsites atun* | THYR.ATUN | 0.022 |
| Echinodermata | Unidentified Echinodermata | ERIZOSI | 0.022 |
| Elasmobranchii | *Centroscymnus macracanthus* | CentroMacra | 0.022 |
| Malacostraca | *Glyphocrangon alata* | GlyphAla | 0.022 |
| Anthozoa | *Alcyonium* spp. | ALCYOSP | 0.022 |
| Teleostei | *Coelorinchus fasciatus* | CoeloFasc | 0.022 |
| Elasmobranchii | *Mustelus mento* | MusteMento | 0.022 |
| Teleostei | Unidentified grenadier | GRANSI | 0.022 |
| Elasmobranchii | *Prionace glauca* | PRIONGL | 0.022 |
| Teleostei | *Seriolella violacea* | SerioViol | 0.022 |
