# Supplementary material for: Spatio-temporal patterns of the crustacean demersal fishery discard from the south Humboldt Current System, based on scientific observer program (2014–2019)
Source: PLoS One. 2023 Feb 27;18(2):e0281932. doi: 10.1371/journal.pone.0281932 (PMC9970059; doi:10.1371/journal.pone.0281932)
Supplement: Attachment — Submitted filename: Responses to reviewers.docx [file pone.0281932.s003.docx]

PONE-D-22-26514

Spatio-temporal patterns of the crustacean demersal fishery discard from the south Humboldt Current System, based on scientific observer program (2014-2019)

PLOS ONE

Dear Dr. Riascos:

Attached you will find the new version of our manuscript PONE-D-22-26514 submitted to PLoS ONE as original article. In this version we have incorporated all the comments and suggestions made by two reviewers, Dr. Paramo and Dr. Yildiz. In the following pages, you will find the responses for every comment made by them, and in the Track changes version, you will find the new additions highlighted in yellow.

We hope this new version will be suitable for publication in PLoS ONE.

Kind regards

Dr. Mauricio F. Landaeta

Professor

Universidad de Valparaíso, Chile

Journal Requirements:

When submitting your revision, we need you to address these additional requirements.

1. Please ensure that your manuscript meets PLOS ONE's style requirements, including those for file naming. The PLOS ONE style templates can be found at <https://journals.plos.org/plosone/s/file?id=wjVg/PLOSOne_formatting_sample_main_body.pdf> and 
<https://journals.plos.org/plosone/s/file?id=ba62/PLOSOne_formatting_sample_title_authors_affiliations.pdf>

2. In your Methods section, please provide additional information regarding the permits you obtained for the work. Please ensure you have included the full name of the authority that approved the field site access and, if no permits were required, a brief statement explaining why

3. Thank you for stating the following in the Acknowledgments Section of your manuscript:

"This work was partially funded by the Millenium Nucleus for Ecology and Conservation of Temperate Mesophotic Reef Ecosystems (NUTME) grant NCN19_056 to APM. We appreciate the comments and suggestions by Dr. P. Saenz-Agudelo (Universidad Austral de Chile)."

We note that you have provided funding information that is not currently declared in your Funding Statement. However, funding information should not appear in the Acknowledgments section or other areas of your manuscript. We will only publish funding information present in the Funding Statement section of the online submission form. 
Please remove any funding-related text from the manuscript and let us know how you would like to update your Funding Statement. Currently, your Funding Statement reads as follows:

"This work was partially funded by the Millenium Nucleus for Ecology and Conservation of Temperate Mesophotic Reef Ecosystems (NUTME) grant NCN19_056 to APM, EAW, SAN, RB, PSA and MFL"

Please include your amended statements within your cover letter; we will change the online submission form on your behalf.

*R. In this new version, we included a Funding Statement, and corrections were made to the Acknowledgements section.*

Please review your reference list to ensure that it is complete and correct. If you have cited papers that have been retracted, please include the rationale for doing so in the manuscript text, or remove these references and replace them with relevant current references. Any changes to the reference list should be mentioned in the rebuttal letter that accompanies your revised manuscript. If you need to cite a retracted article, indicate the article’s retracted status in the References list and also include a citation and full reference for the retraction notice.

[Note: HTML markup is below. Please do not edit.]

Reviewers' comments:

Reviewer's Responses to Questions

**Comments to the Author**

1. Is the manuscript technically sound, and do the data support the conclusions?

The manuscript must describe a technically sound piece of scientific research with data that supports the conclusions. Experiments must have been conducted rigorously, with appropriate controls, replication, and sample sizes. The conclusions must be drawn appropriately based on the data presented.

Reviewer #1: Yes

Reviewer #2: Yes

2. Has the statistical analysis been performed appropriately and rigorously?

Reviewer #1: Yes

Reviewer #2: Yes

3. Have the authors made all data underlying the findings in their manuscript fully available?

The [PLOS Data policy](http://www.plosone.org/static/policies.action#sharing) requires authors to make all data underlying the findings described in their manuscript fully available without restriction, with rare exception (please refer to the Data Availability Statement in the manuscript PDF file). The data should be provided as part of the manuscript or its supporting information, or deposited to a public repository. For example, in addition to summary statistics, the data points behind means, medians and variance measures should be available. If there are restrictions on publicly sharing data—e.g. participant privacy or use of data from a third party—those must be specified.

Reviewer #1: Yes

Reviewer #2: Yes

4. Is the manuscript presented in an intelligible fashion and written in standard English?

PLOS ONE does not copyedit accepted manuscripts, so the language in submitted articles must be clear, correct, and unambiguous. Any typographical or grammatical errors should be corrected at revision, so please note any specific errors here.

Reviewer #1: Yes

Reviewer #2: Yes

5. Review Comments to the Author

Please use the space provided to explain your answers to the questions above. You may also include additional comments for the author, including concerns about dual publication, research ethics, or publication ethics. (Please upload your review as an attachment if it exceeds 20,000 characters)

**Reviewer #1:** The manuscript is very interesting because it analyzes the spatio-temporal patterns of the crustacean demersal fishery discard from the south Humboldt Current System, based on scientific observer program. Some corrections and analysis are required before being accepted to publish.

Line 69: change ... demersal crustacean fisheries by ... demersal crustacean chilean fisheries.

*Resp. The change was done accordingly.*

Delete table 1 and replace with a species accumulation curves using the samples-based rarefaction method for each year.

*R. Table 1 was deleted following the suggestions by the reviewer. Also, we carried out the species accumulation curves by year.*

Line 127: Differences between industrial and artisanal fleets are also related to engine power and fishing gear dimensions. Please evaluate if there are differences between the industrial and artisanal fleets with respect to engine power and the dimensions of the fishing gear.

*R. In this version, we included the mean engine power of each fleet, including a statement indicating that the fishing gear was the same in dimensions for artisanal and industrial fleets.*

Figure 3: correct the names in the X and Y axis that are cut off.

*R. Figure 3, now Figure 4, was corrected and improved.*

**Reviewer #2**: The article evaluates detailed data on data collection by observers in the problem of discard, which is an important problem in fisheries. The applied statistics were chosen correctly. however, some additions are required for a more understandable flow. The parts that need to be added are indicated on the text.

Line 126: commercial bottom trawler??? please clarify!

*R. the explanation was included in the new version.*

Line 123: please provide more informtion about the temporal scale (open period, closed season etc) of the fishery(ies)!

*R. We included a new sentence “The Chilean crustacean fisheries are open throughout the year, except for September, when a reproductive ban occurs, since 2015.”*

Line 127: I wonder if a metier analysis has been made for the region, can you give some information?

*R. Until now, only a metier analysis has been made recently for the cardinalfish (Epigonus crassicaudus) fishery (Cubillos et al. 2022). However, those type of analysis still is lacking for the Chilean demersal crustacean fisheries.*

Line 127: specify the fishing gear?

*R. Now, we have included specifications of the fishing gear.*

Line 127: use same mesh size in the cod-end? if they use bottom trawl?

*R. Yes, all the fleet uses the same gear and mesh size, according to Chilean laws. This information is given in the new version of the ms.*

Line 129: I wonder if there is a trend related to the daily vertical distribution of Merluccius gayi regarding night hauls, because it will definitely show itself in the discard.

*R. Hauls were made during night (21:00 to 02:00 h) and day hours (09:00 to 20:00 hrs), but irrespective of the time of the day, the occurrence of M. gayi in the hauls was always high (95%).*

Line 129: Can you provide information about haul duration such as minimum and maximum values?

*R. This information (minimum, maximum, mean, one SD) was included in the new version.*

Line 138: Who decided to discard frction of catch, fishers? please specified!

*R. According to the Chilean laws, only the catches of the target species can be landed, and therefore, non-target species are discarded, e.g., thrown away at sea.*

Line 138: These species are always discarded?

*R. Yes, always.*

Line 139: please provide more detailed information about this program and detail the observation processor!

*R. We included a paragraph with a link to a web page, where there is more detailed information about this program, leaded by Instituto de Fomento Pesquero (IFOP, Chile).*

Line 202: Please specify which program is used for gam modelling.

*R. We included this information in the new version of the ms.*

Line 208: why did you work with different distribution families at this point?

*R. We used the Poisson distribution to model the richness of species (counting follows a Poisson distribution), while the Gaussian distribution was used to model the other diversity indices (it is supposed that the error structure follows a normal distribution).*

6. PLOS authors have the option to publish the peer review history of their article ([what does this mean?](https://journals.plos.org/plosone/s/editorial-and-peer-review-process#loc-peer-review-history)). If published, this will include your full peer review and any attached files.


If you choose “no”, your identity will remain anonymous but your review may still be made public.


**Do you want your identity to be public for this peer review?** For information about this choice, including consent withdrawal, please see our [Privacy Policy](https://www.plos.org/privacy-policy).

Reviewer #1: **Yes:** Jorge Paramo, Universidad del Magdalena, Colombia

Reviewer #2: **Yes:** Taner Yildiz
